# Genetically Determined Circulating Levels of Cytokines and the Risk of Rheumatoid Arthritis

**DOI:** 10.3389/fgene.2022.802464

**Published:** 2022-02-07

**Authors:** Yu Qian, Zhixing He, Sizheng Steven Zhao, Bin Liu, Ying Chen, Xiaohui Sun, Ding Ye, Xia Jiang, Hui Zheng, Chengping Wen, Houfeng Zheng, Yingying Mao

**Affiliations:** ^1^ Fudan University, Shanghai, China; ^2^ Diseases and Population (DaP) Geninfo Lab, School of Life Sciences, Westlake University, Hangzhou, China; ^3^ Department of Epidemiology, School of Public Health, Zhejiang Chinese Medical University, Hangzhou, China; ^4^ School of Basic Medical Sciences, Zhejiang Chinese Medical University, Hangzhou, China; ^5^ Musculoskeletal Biology, Institute of Lifecourse and Medical Sciences, University of Liverpool, Liverpool, United Kingdom; ^6^ Center for Molecular Medicine, Karolinska Institute, Stockholm, Sweden; ^7^ Department of Epidemiology, Harvard T.H. Chan School of Public Health, Boston, MA, United States; ^8^ Institute of Biology and Medical Sciences, Soochow University, Suzhou, China

**Keywords:** cytokine, genome-wide association study, macrophage inflammatory protein-1β, mendelian randomization, rheumatoid arthritis, colocalization analysis

## Abstract

**Background:** Accumulation of inflammatory leukocytes in articular tissues is the hallmark feature of rheumatoid arthritis (RA). Increasing evidence from observational studies has suggested that several cytokines may be involved in the development of RA. However, traditional observational studies are susceptible to bias from confounding and reverse causation; therefore, the potential causal relationships of individual cytokines with the risk of RA remain elusive.

**Objective:** In this study, we evaluated whether genetically determined circulating levels of cytokines were associated with the risk of RA by performing Mendelian randomization (MR).

**Methods:** We identified single nucleotide polymorphisms (SNPs) associated with circulating levels of cytokines and growth factors from a genome-wide association study (GWAS) including 8,293 participants of Finnish ancestry as instrumental variables (IVs). The association estimates of these IVs with the risk of RA were obtained from a GWAS meta-analysis including 14,361 RA cases and 43,923 controls of European ancestry. We conducted a series of MR analyses to assess the relationship between genetically determined circulating cytokines and the risk of RA, including the random-effects inverse variance-weighted, weighted-median, MR-Egger regression, and MR pleiotropy residual sum and outlier tests. For potential cytokine-RA associations supported by MR evidence, sensitivity analyses were further performed using restricted IV sets of SNPs with colocalization evidence and that excluding pleiotropic SNPs.

**Results:** In the primary MR analysis, there was a suggestive inverse association between genetically determined circulating level of macrophage inflammatory protein-1β (MIP-1b) and the risk of RA [odds ratio (OR): 0.95, 95% confidence interval (CI) = 0.92-0.99, *p* = 0.016]. The effect estimates were similar in alternative MR analyses. Among SNPs used as IVs for MIP-1b, we found 92 SNPs without documented pleiotropy and three SNPs with evidence of colocalization. The association of MIP-1b with RA from sensitivity analyses using these two sets of restricted IVs remained stable.

**Conclusion:** Our study suggests that genetically determined elevated circulating level of MIP-1b may be associated with a lower risk of RA. Further studies are warranted to determine how MIP-1b and related pathways may contribute to the development of RA.

## 1 Introduction

Rheumatoid arthritis (RA) is a chronic systemic inflammatory disease affecting approximately 1% of the global population (Smolen et al., 2018). Lymphocytic promotion of auto-antibodies and inflammatory cytokines are central to its pathology. Genome-wide association studies (GWASs) have confirmed the high heritability of RA (∼65%) (MacGregor et al., 2000), and RA-associated single nucleotide polymorphisms (SNPs) enrichment in or related to genes active in CD4^+^ T cells (Okada et al., 2014). Improved understanding in these areas has translated to successful therapies that have revolutionized its treatment. Targeted immune therapies, such as tumor necrosis factor (TNF) inhibitors, have been widely used in RA treatment (McInnes and Schett, 2017). However, less than a quarter of patients achieve remission at 6 months (Hetland et al., 2010), and up to 10% remain “resistant” to multiple conventional synthetics and biologic disease-modifying antirheumatic drugs (DMARDs) (de Hair et al., 2018). Alternative cytokine-pathways (and therefore potential therapies) may be relevant in these patients.

Accumulation of inflammatory leukocytes in articular tissues is the hallmark feature of RA. Why articular tissues are preferentially affected, and the process of cellular recruitment into the inflamed tissue, remains unclear. Increasing evidence from observational studies has suggested that cytokines, such as monocyte chemoattractant protein-1 and interleukin-1 α, might be involved in the disease process (Rantapää-Dahlqvist et al., 2007; Jorgensen et al., 2008). However, conventional observational studies are susceptible to bias from confounding and reverse causation; therefore, the potential causal relationships of individual cytokines with the risk of RA remain elusive.

Mendelian randomization (MR) is a method that uses genetic variants as instrumental variables (IVs) for the exposures and assesses their associations with disease outcomes (Davey Smith and Ebrahim, 2003). Unlike traditional observational epidemiological studies, the use of MR analyses can reduce biases, such as reverse causation (because genetic variants are not affected by diseases) and residual confounding (because alleles are randomly assigned to offsprings during conception). When the three key assumptions (i.e., first genetic variants used as IVs are associated with the exposure of interest; second these IVs are not associated with confounders or other disease outcomes; and third these genetic variants affects the outcome only through the exposure of interest) are satisfied, MR analyses can provide clues for a potential causal association between the exposure and the outcome (Davey Smith and Ebrahim, 2003). Additionally, colocalization analysis can be used to investigate whether the association between circulating protein and RA was driven by the same genetic variants across these phenotypes or confounded by linkage disequilibrium (LD) (i.e. genetic variants correlation) (Giambartolomei et al., 2014). Hence, the combination of MR and colocalization enables the exploration of associations for which there is a paucity of observational evidence, as is the case for cytokines and RA.

In this study, we systematically evaluated the relationships between circulating levels of cytokines and the risk of RA by conducting a two-sample MR study in a large GWAS meta-analysis of 14,361 participants with RA and 43,923 control participants (Okada et al., 2014). Sensitivity analyese were further performed based on pleiotropy search and colocalization evidence to test the validity of our results.

## 2 Methods

### 2.1 Study Design

**FIGURE 1:**
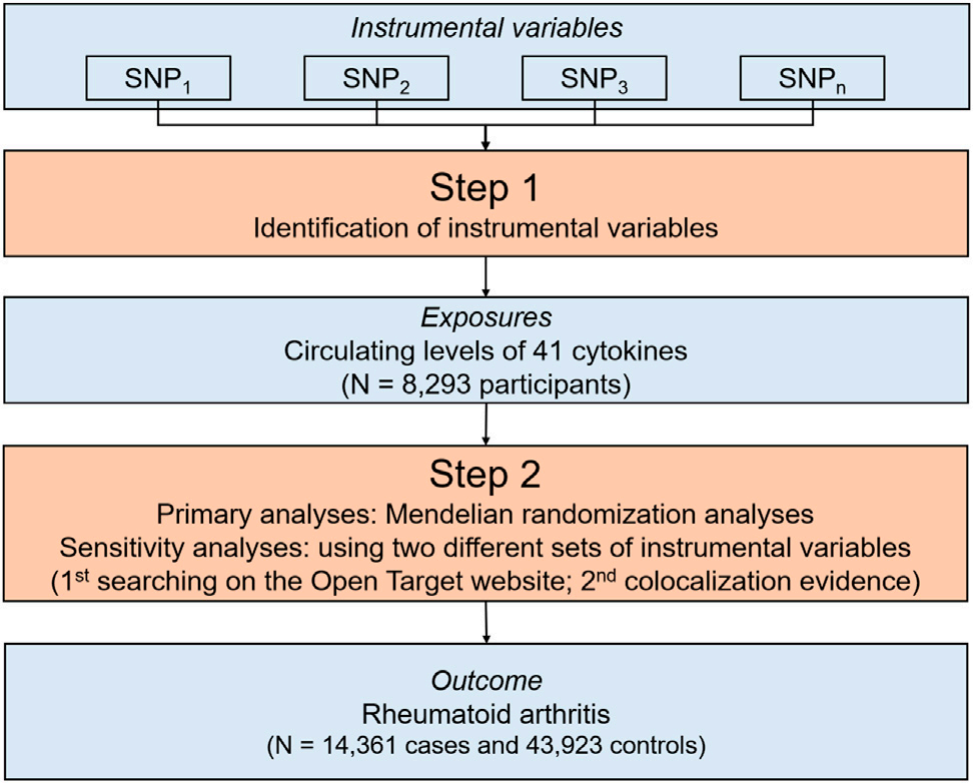
An overall design of the present study. Abbreviations: MR, Mendelian randomization; N, number; SNP, single nucleotide polymorphism.

The overall design of the study is summarized in Figure 1. Briefly, we first identified SNPs associated with circulating levels of cytokines to be used as IVs from summary statistics of 8,293 participants of Finnish ancestry (Ahola-Olli et al., 2017). Then, we performed a series of MR analyses to explore the association between circulating levels of individual cytokines and the risk of RA using the summary statistics including 58,284 participants of European ancestry (Okada et al., 2014). For potential cytokine-RA associations supported by MR evidence, we further conducted a series of sensitivity analyses using two different IV sets. Briefly, we scanned each SNPs used as IVs for their potential secondary phenotypes, and performed sensitivity analyses using updated IVs excluding pleiotropic SNPs. Additionally, we conducted colocalization analyses, and then re-run the MR analyses using SNPs with colocalization evidence. Since all the analyses were based on publicly available summary statistics, no additional ethical approval or consent to human participants was required.

### 2.2 Source of Genetic Summary Statistics

The detailed characteristics of the GWAS summary statistics used in our MR analyses, including quality control, have been previously described elsewhere (Okada et al., 2014; Ahola-Olli et al., 2017). In Supplementary Table S1, we provided related information and the links for downloading these statistics. Briefly, summary statistics for RA were obtained from a GWAS meta-analysis including 14,361 participants with RA and 43,923 control participants of European ancestry (Okada et al., 2014). All RA cases met the 1987 RA-related diagnostic criteria of the American College of Rheumatology, or were diagnosed by a board-certified rheumatologist (Okada et al., 2014). For quality control, individuals with more than 1–5% missing genotypes were excluded, and variants with a minor allele frequency <0.5% and those with a *p*-value for Hardy–Weinberg equilibrium ≤1.0 × 10^–6^ in all samples were also excluded (Okada et al., 2014). For summary statistics of circulating cytokines, a total of 10.7 million genetic variants with imputation info ≥0.7, model fit info ≥0.7, and minor allele count ≥10 were included in the GWAS meta-analyses including 8,293 Finnish participants (Ahola-Olli et al., 2017).

### 2.3 Selection of SNPs

We selected SNPs reaching the genome-wide level of significance (*p* < 5 × 10^−8^) for their associations with circulating levels of cytokines. Then, for each cytokine, SNPs used as IVs were pruned for LD at a stringent threshold (*r* ^2^ < 0.1), and those with a larger *p-*value for the exposure of interest were excluded. We identified a total of 958 SNPs associated with circulating levels of 28 cytokines. We further excluded SNPs for which associations of these SNPs and their proxies (SNPs correlated at *r* ^2^ > 0.9) were not available in RA summary statistics, leaving a total of 270 SNPs (including one proxy) as the final IV sets for the 27 circulating cytokines (Supplementary Table S2).

In sensitivity analyses, we used several alternative sets of IVs. Specifically, to minimize the effect of potential pleiotropic SNPs on causal association estimates, we excluded pleiotropic SNPs showing associations of *p* < 5 × 10^−8^ with other traits using the Open Targets (Last accessed on 9 October 2021). Additionally, a restricted IV set were constructed including independent SNPs with colocalization evidence.

### 2.4 Statistical Analysis

#### 2.4.1 MR Analyses

We assessed the relationships between these circulating cytokines and the risk of RA using two-sample MR approach. After extraction of the association estimates of SNP-cytokine and SNP-RA and harmonization of the direction of estimates by effect alleles, we estimated the proportions of variances in circulating levels of cytokines explained by our primary IVs (*R* ^2^) (Park et al., 2010). *F*-statistics were calculated to quantify the strength of the IVs using the method previously described (Burgess et al., 2011). We then computed individual MR estimates with the Wald estimator and standard error with the Delta method (Burgess et al., 2013). We performed Cochran’s Q test to assess the heterogeneity among IVs used for each circulating cytokine, and pooled these individual estimates using the random-effects inverse-variance-weighted (IVW) method (Burgess et al., 2013) as primary analysis. To assess the robustness of the association estimates, we further performed alternative MR analyses including the weighted-median method, MR-Egger regression, and MR pleiotropy residual sum and outlier (MR-PRESSO) test. Specifically, the weighted-median method can provide a valid estimate under the assumption that less than 50% of the weights are invalid (Bowden et al., 2016). Additionally, the intercept of the MR-Egger regression can be used as an indicator of directional pleiotropy (*p*-value for intercept <0.05 indicated the presence of directional pleiotropy) (Bowden et al., 2015). The possible pleiotropic outliers can also be identified using the MR-PRESSSO test, and outliers were excluded from the primary genetic IVs and the causal effect estimates were reassessed using the remaining IVs (Verbanck et al., 2018).

#### 2.4.2 Colocalization Analyses

We further included SNPs within ±1 megabase region of the SNPs associated with circulating levels of cytokines at genome-wide significance level to form candidate SNP sets. Then, we extracted the association estimates of these SNPs with the risk of RA. Based on the assumption that there was one genetic variant within each region shared across two phenotypes (i.e., circulating cytokine level and RA), the Bayesian framework was used to test the posterior probability of association (PPA) for four hypotheses, according to default colocalization protocols (Giambartolomei et al., 2014). For SNPs with PPA for the fourth hypothesis (i.e., both traits were associated and share a single causal genetic variant) > 0.95, it was considered as having strong evidence of colocalization (Giambartolomei et al., 2014).

We corrected for multiple comparisons using Bonferroni approach and set statistical significance at a *p*-value < 0.002 (0.05/27) based on the number of cytokines included in the primary MR analyses. *p-*value above 0.002 but less than 0.05 were considered suggestive evidence for an observed association. All analyses were performed in R software (v 3.6.2) using coloc (Wallace et al., 2012), MendelianRandomization (Yavorska and Burgess, 2017), and MRPRESSO packages (Verbanck et al., 2018).

## 3 Results

**FIGURE 2:**
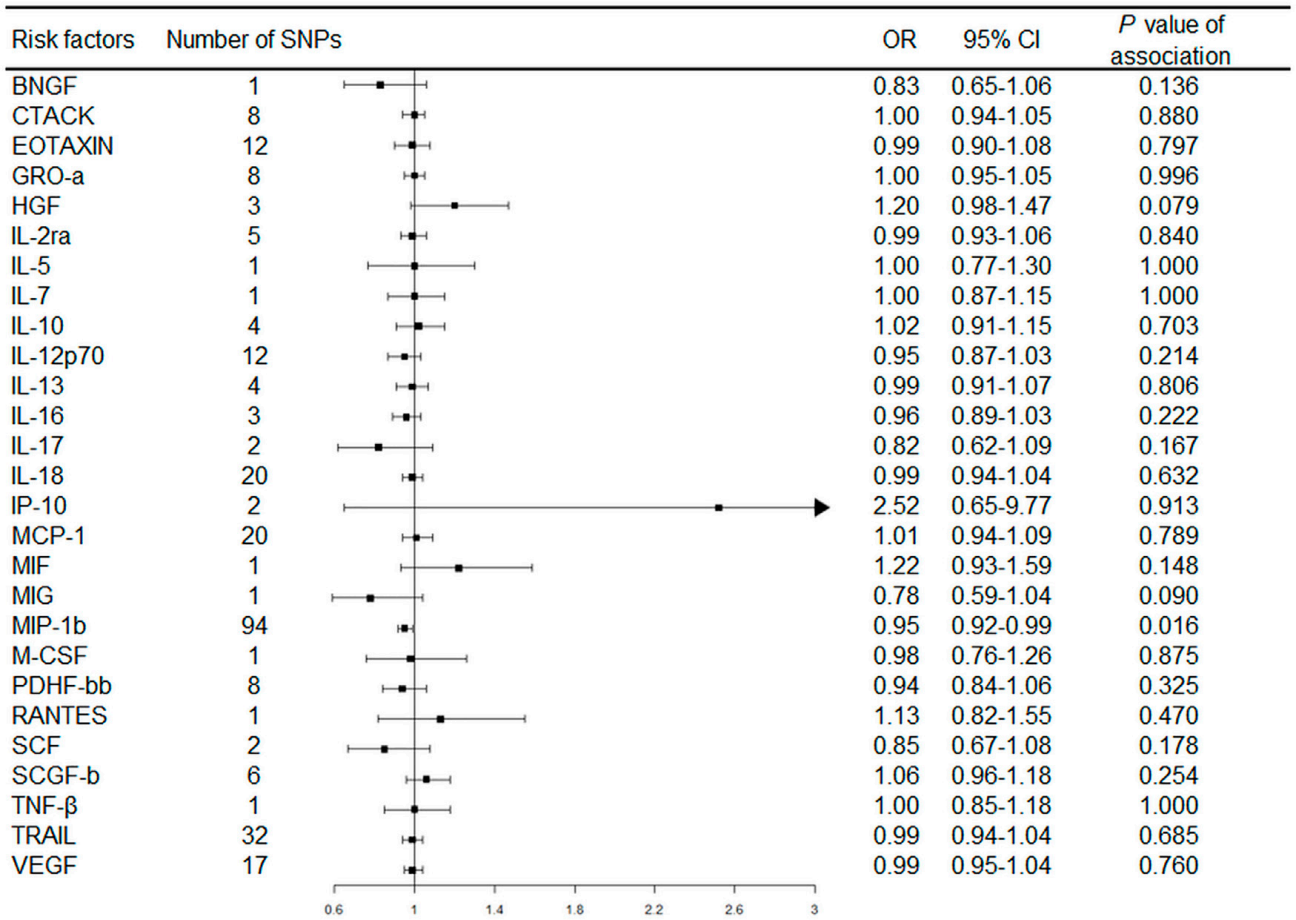
Forest plot of Mendelian randomization analysis for the associations between circulating cytokines with rheumatoid arthritis risk. Abbreviations: CI, confidence interval; OR, odds ratio; SNP, single nucleotide polymorphism.

**FIGURE 3:**
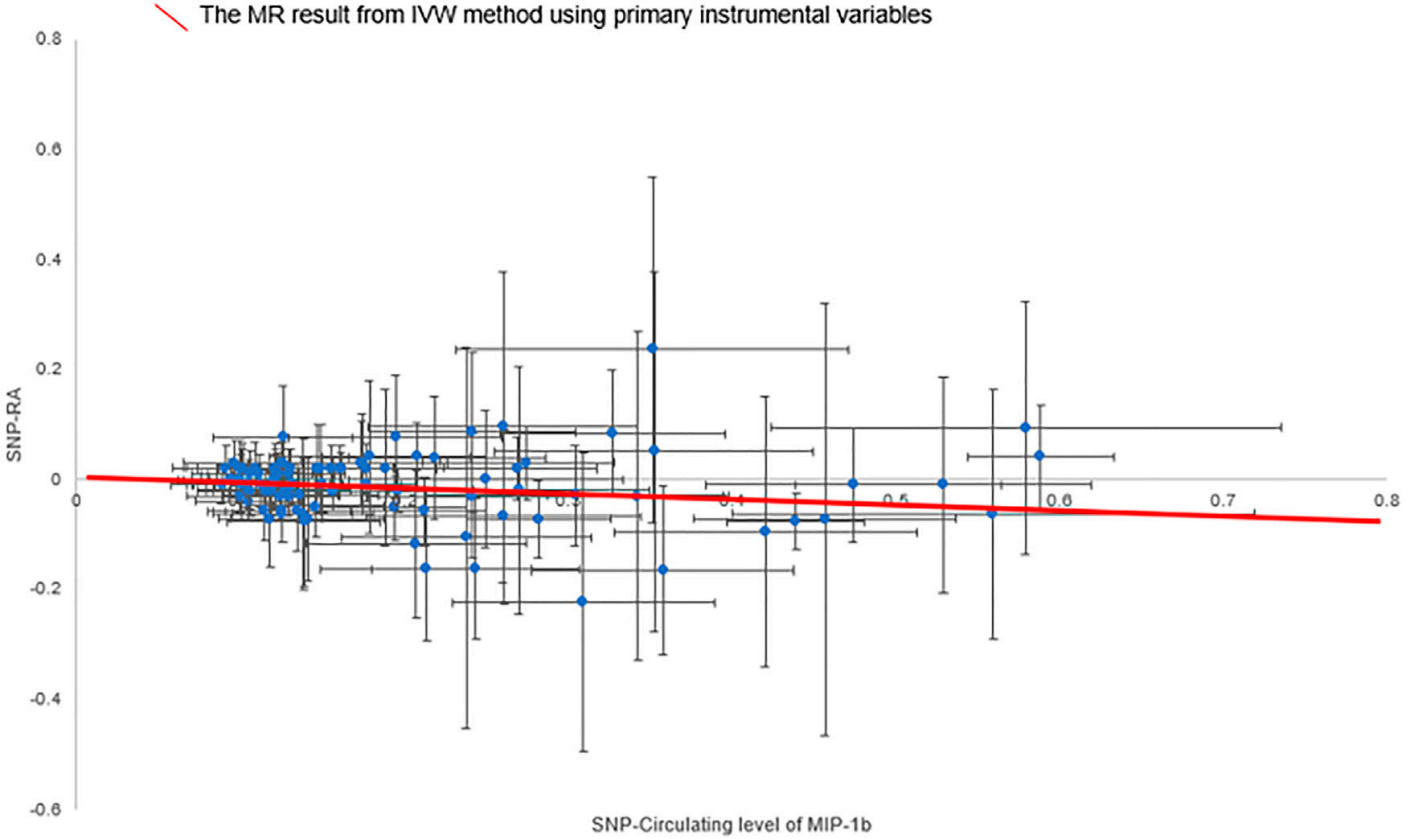
Scatter plot of the associations of genetic variants with circulating level of MIP-1b and the risk of rheumatoid arthritis. Abbreviations: IVW, inverse-variance weighted; MIP-1b, macrophage inflammatory protein-1β; MR, Mendelian randomization; SNP, single nucleotide polymorphism; RA, rheumatoid arthritis.

The primary MR results from the random-effects IVW method are presented in Figure 2. Overall, after Bonferroni correction for multiple comparisons, there were no circulating levels of cytokines showing statistically significant associations with risk of RA (*p*-value < 0.002). Notably, circulating levels of macrophage inflammatory protein-1β (MIP-1b/CCL4) showed suggestive inverse association with the risk of RA [odds ratio (OR): 0.95, 95% confidence interval (CI) = 0.92-0.99, *p* = 0.016] using the random-effects IVW method (Figure 3). The detailed information of the IVs used for MIP-1b is listed in Supplementary Table S3.

In addition, in the weighted-median method, the association of MIP-1b with the risk of RA remained stable, though with wider confidence intervals (OR: 0.94, 95% CI = 0.88-0.99, *p* = 0.034). Moreover, there was no evidence showing the presence of directional pleiotropy effects as assessed by MR-Egger regression (*P* for intercept = 0.859), and no outliers were detected using the MR-PRESSO test (Supplementary Table S4).

In sensitivity analyses using alternative IV sets, we first systemically scanned individual SNPs used as IVs for MIP-1b for potential pleiotropy. We found that no SNPs were associated with any other cytokines at a genome-wide significance level, but there were two pleiotropic variants, based on the Open Targets website (i.e., rs225285: *p*-value for highest math class taken = 3.0 × 10^−10^ (Lee et al., 2018); and rs2073495: *p*-value for monocyte count = 5.5 × 10^−12^ (Chen et al., 2020)). After excluding these two SNPs, the association estimate of genetically determined circulating level of MIP-1b with the risk of RA remained consistent (OR: 0.95, 95% CI = 0.91-0.99, *p* = 0.017).

Furthermore, based on the observed MR evidence, we conducted colocalization analysis of RA and MIP-1b. We found a total of 10 SNPs with colocalization evidence for circulating MIP-1b and RA (Supplementary Table S5). After pruned in LD (*r* ^2^ < 0.1), three independent non-pleiotropic SNPs remained in the final IV set. MR analyses based on this restricted IV set yielded a similar association between circulating MIP-1b and the risk of RA (OR: 0.76, 95% CI = 0.69-0.84, *p* = 1.73 × 10^−7^ for fixed-effects IVW method).

## 4 Discussion

By using two-sample MR and colocalization, we systematically assessed the potential causal relationships between circulating levels of cytokines and the risk of RA. Our study found a suggestive inverse association between genetically determined circulating level of MIP-1b and the risk of RA. Moreover, the results for the inverse association were stable using alternative MR methods and in sensitivity analyses using alternative IV sets. Collectively, these findings provided evidence for a potential protective role of circulating MIP-1b in the development of RA.

Results of the association between MIP-1b and RA from previous observational epidemiological studies were inconsistent. For example, a case-control study including 43 untreated early RA patients and 14 healthy controls found that plasma levels of MIP-1b were higher in RA patients than in healthy controls (*p* < 0.01) (Pandya et al., 2017). In another case-control study including 14 RA patients and 27 controls, though there were no differences in serum level of MIP-1b in RA patients compared with healthy controls [log transformed level (range):4.92 (4.19–5.89) vs 4.74 (3.14-5.72)] (Barra et al., 2014), serum MIP-1β was negatively associated with anticitrullinated protein/peptide antibodies (OR: 0.03; 95% CI = 0.01–0.39; *p* = 0.007) (Barra et al., 2014), which are established biomarkers for RA diagnosis and can predict radiographic joint damage (Taylor et al., 2011). Differences in these findings may be explained by several reasons. First, these observational studies included smaller samples of RA cases and controls, which might result in inadequate statistical power. In addition, the observed associations are susceptible to biases inherent in the observational study design, such as confounding and reverse causality. Our MR study utilized the summary data from published GWAS meta-analysis with the largest sample sizes of RA patients and controls to date including more than 58,000 participants, with an estimated F-statistic of 66.07 to overcome the weak instruments bias and achieve sufficient statistical power (91.84%). Moreover, as genetic variants are presumed to be randomly allocated during meiosis and unaffected by the onset of diseases, confounding factors are anticipated to be equally distributed among different genotypes. Therefore, MR study is more reliable in causal inference, since it minimizes the influence of reverse causation and confounding (Davey Smith and Ebrahim, 2003).

One fundamental assumption for MR study to ensure a valid causal estimate is that genetic variants used as IVs did not directly act on the target outcome, neither affect the outcome through pathways other than through the exposure (Davey Smith and Ebrahim, 2003). To verify this model assumption and to guarantee the validity of results, we first performed a series of MR analyses, such as the weighted-median, MR-Egger regression, and MR-PRESSO tests. The results obtained from these analyses were robust and did not provide evidence of potential pleiotropy. In addition, we manually scanned the SNPs used as IVs for the circulating level of MIP-1b for their potential associations with secondary traits. We found that none of them are associated with RA at a genome-wide significance level, and the causal estimate remained stable in the sensitivity analysis using the restricted IVs excluding pleiotropic SNPs, suggesting the robustness of our findings.

The underlying biological mechanism of MIP-1b in the pathogenesis of RA remains unclear. One possible explanation has been suggested is that MIP-1b is involved in the regulation of Th1 cell trafficking (Patel et al., 2001). Evidence from a case-control study of 24 RA patients and 22 controls showed that the mean (±SEM) level of MIP-1b was higher in the synovial fluids of RA patients (738 ± 282 pg/ml), while it was lower in the serum of RA patients (189 ± 23 pg/ml), as compared to controls (39 ± 6 pg/ml in synovial fluids; 244 ± 68 pg/ml in serum) (Patel et al., 2001). The concentration of MIP-1b in synovial tissues and fluids was elevated to traffic Helper T 1 cells into inflamed synovium (Patel et al., 2001), thus contributed to the development of RA by inducing osteoclastogenesis (Kotake et al., 2005). Further studies were warranted to clarify the potential mechanism of circulating MIP-1b in the pathogenesis of RA.

There were several limitations to this study. First, our results may not be generalizable to other populations, since the ancestry of participants included in our MR study was restricted to Europeans. Second, the effects of genetic variations (e.g. elevated MIP-1b level due to *CCL4* gene polymorphisms) on normal development may influence the expression of other genes, which in turn compensate for the influence of lifelong higher circulating level of MIP-1b (Davey Smith and Ebrahim, 2003). Hence, the likelihood that the MR association reflecting the true potential causal effect might be reduced. Third, since the IVs for several cytokines were not available and the statistical power of genetic associations for some cytokines might be insufficient; it is possible that we might miss potential weak associations of these cytokines with the risk of RA. Further individual level-based MR studies with a prospective design are needed to address these issues. Finally, due to the diversity between Finnish and European population, it is true that it might bring bias because the data from the “exposure” and the “outcome” are of different study populations, but every two-sample MR study may suffer from this.

## 5 Conclusion

Our MR study found suggestive evidence that genetically determined elevated circulating level of MIP-1b was associated with a reduced risk of RA. More studies on the potential biological mechanism underlying MIP-1b and risk of RA are warranted to explore whether MIP-1b or related pathways could be potential therapeutic targets in the prevention of RA.

## Data Availability

The original contributions presented in the study are included in the article/Supplementary Material, further inquiries can be directed to the corresponding authors.
