# Supplementary material for: Genetically Determined Circulating Levels of Cytokines and the Risk of Rheumatoid Arthritis
Source: Front Genet. 2022 Feb 7;13:802464. doi: 10.3389/fgene.2022.802464 (PMC8859847; doi:10.3389/fgene.2022.802464)
Supplement: Supplementary file 1 [file DataSheet1.docx]

Supplementary Material

**Supplementary Table 1** Details of the genome-wide association studies and datasets used in our analyses.

**Supplementary Table 2** Characteristics of the instrumental variables used for the 27 cytokines. ^*^Total variance was calculated in an additive model assuming no interaction between the individual single nucleotide polymorphisms.

**Supplementary Table 3** Characteristics of the SNPs associated with circulating level of MIP-1b and their associations with RA. Abbreviations: CCL-4, C-C chemokine ligand 4; MIP-1b, macrophage inflammatory protein-1β; RA, rheumatoid arthritis; SNPs, single nucleotide polymorphisms.

**Supplementary Table 4** Primary MR analyses for the association between circulating MIP-1b level and RA risk. ^*^*P*-value of the intercept from MR-Egger regression analysis. Abbreviations: CI, confidence interval; MIP-1b, macrophage inflammatory protein-1β; MR, Mendelian randomization; MR-PRESSO test, MR Pleiotropy RESidual Sum and Outlier test; OR, odds ratio; RA, rheumatoid arthritis; SNP, single nucleotide polymorphism.

**Supplementary Table 5** The detailed information of SNPs with colocalization evidence among IVs used for MIP-1b. Abbreviations: IV, instrumental variable; MIP-1b, macrophage inflammatory protein-1β; SNP, single nucleotide polymorphism.

| **Supplementary Table 1.** Details of the genome-wide association studies and datasets used in our analyses. | | | | |
| --- | --- | --- | --- | --- |
| Exposure  or outcome | Sample size | Ancestry | Links for data download | PMID |
| Circulating levels of 41 cytokines | 8293 participants | Finnish | https://www.ebi.ac.uk/gwas/publications/27989323 | 27989323 |
| Rheumatoid arthritis | 14,361 cases and 43,923 controls | European | <http://plaza.umin.ac.jp/~yokada/datasource/software.htm> | 24390342 |

**Supplementary Table 2** Characteristics of the instrumental variables used for the 27 cytokines

| Cytokines | No. of primary  instrumental variables | Variance explained by the | *F*-statistic of the primary  instrumental variables |
| --- | --- | --- | --- |
|  |  | primary instrumental |  |
|  |  | variables (R^2^)^*^ |  |
| BNGF | 1 | 0.011 | 91.48 |
| CTACK | 8 | 0.091 | 103.87 |
| Eotaxin | 12 | 0.061 | 45.03 |
| GRO-a | 8 | 0.291 | 424.25 |
| HGF | 3 | 0.016 | 45.22 |
| IL-2ra | 5 | 0.08 | 143.78 |
| IL-5 | 1 | 0.011 | 94.51 |
| IL-7 | 1 | 0.052 | 452.29 |
| IL-10 | 4 | 0.056 | 122.95 |
| IL-12p70 | 12 | 0.129 | 102.48 |
| IL-13 | 4 | 0.119 | 279.56 |
| IL-16 | 3 | 0.163 | 537.32 |
| IL-17 | 2 | 0.006 | 24.31 |
| IL-18 | 20 | 0.182 | 91.91 |
| IP-10 | 2 | 0.041 | 178.59 |
| MCP-1 | 20 | 0.08 | 35.78 |
| MIF | 1 | 0.012 | 101.22 |
| MIG | 1 | 0.009 | 75.71 |
| MIP-1b | 94 | 0.431 | 66.07 |
| M-CSF | 1 | 0.007 | 55.96 |
| PDGF-bb | 8 | 0.061 | 66.82 |
| RANTES | 1 | 0.002 | 14.09 |
| SCF | 2 | 0.011 | 44.09 |
| SCGF-b | 6 | 0.054 | 78.3 |
| TNF-β | 1 | 0.104 | 967.16 |
| TRAIL | 32 | 0.271 | 95.8 |
| VEGF | 17 | 0.281 | 189.85 |

^*^Total variance was calculated in an additive model assuming no interaction between the individual single nucleotide polymorphisms.

| **Supplementary Table 3** Characteristics of the SNPs associated with circulating level of MIP-1b and their associations with RA | | | | | | | | | | | |
| --- | --- | --- | --- | --- | --- | --- | --- | --- | --- | --- | --- |
| SNP | Chr | Position | MIP-1b | | | | Rheumatoid arthritis | | | | Whether pleiotropic |
|  |  |  | Effect allele | beta | se | *P*-value | Effect allele | beta | se | *P*-value |  |
| rs73072664 | 3 | 44526642 | C | -0.26 | 0.042 | 5.46E-10 | A | 0.095 | 0.144 | 0.5 | No |
| rs114164513 | 3 | 45272343 | C | 0.327 | 0.035 | 2.09E-20 | T | -0.083 | 0.058 | 0.13 | No |
| rs79068918 | 3 | 45344715 | G | -0.275 | 0.027 | 3.20E-24 | G | -0.03 | 0.034 | 0.43 | No |
| rs62243190 | 3 | 45524938 | C | -0.457 | 0.041 | 4.17E-29 | T | -0.073 | 0.2 | 0.71 | No |
| rs191600590 | 3 | 45610258 | T | -0.15 | 0.025 | 8.79E-10 | A | -0.01 | 0.026 | 0.65 | No |
| rs2673050 | 3 | 45739807 | G | -0.131 | 0.016 | 3.14E-16 | T | -0.01 | 0.018 | 0.75 | No |
| rs73062378 | 3 | 45822010 | C | 0.212 | 0.019 | 4.43E-28 | T | 0.058 | 0.031 | 0.04 | No |
| rs4683146 | 3 | 45898221 | G | -0.177 | 0.016 | 1.45E-29 | A | -0.01 | 0.015 | 0.41 | No |
| rs12630732 | 3 | 45909682 | T | 0.121 | 0.019 | 2.13E-10 | A | 0.01 | 0.02 | 0.73 | No |
| rs150648632 | 3 | 45909682 | TAA | 0.112 | 0.017 | 3.15E-11 | A | 0.01 | 0.02 | 0.73 | No |
| rs4683154 | 3 | 45979280 | T | -0.128 | 0.016 | 5.95E-16 | A | -0.02 | 0.016 | 0.24 | No |
| rs7610953 | 3 | 46043436 | G | 0.122 | 0.018 | 3.42E-11 | G | 0.01 | 0.018 | 0.64 | No |
| rs190685138 | 3 | 46063688 | G | -0.56 | 0.082 | 6.51E-12 | A | -0.062 | 0.116 | 0.59 | No |
| rs974381 | 3 | 46074554 | T | -0.111 | 0.018 | 7.08E-10 | A | 0.01 | 0.018 | 0.58 | No |
| rs35178150 | 3 | 46194769 | G | 0.126 | 0.022 | 5.38E-09 | A | 0.058 | 0.029 | 0.04 | No |
| rs35960950 | 3 | 46199680 | G | 0.126 | 0.022 | 4.80E-09 | G | -0.062 | 0.027 | 0.03 | No |
| rs1542754 | 3 | 46272456 | C | -0.156 | 0.018 | 7.36E-19 | T | -0.02 | 0.018 | 0.39 | No |
| rs189356071 | 3 | 46284456 | G | -0.131 | 0.023 | 1.01E-08 | A | 0.02 | 0.018 | 0.37 | No |
| rs138778453 | 3 | 46292810 | C | 0.105 | 0.016 | 9.97E-11 | T | 0.039 | 0.017 | 0.01 | No |
| rs76399503 | 3 | 46472207 | G | 0.122 | 0.02 | 1.96E-09 | A | 0 | 0.028 | 0.99 | No |
| rs9110 | 3 | 46480801 | G | -0.116 | 0.017 | 2.37E-12 | A | -0.02 | 0.018 | 0.28 | No |
| rs2073495 | 3 | 46480958 | G | -0.119 | 0.018 | 2.47E-11 | G | 0.01 | 0.018 | 0.59 | Yes |
| rs7609565 | 3 | 46482683 | T | 0.146 | 0.021 | 4.72E-12 | A | 0.049 | 0.029 | 0.13 | No |
| rs2230894 | 3 | 46490474 | G | 0.13 | 0.019 | 5.78E-12 | A | 0.02 | 0.023 | 0.44 | No |
| rs4683228 | 3 | 46498261 | C | 0.105 | 0.016 | 4.30E-11 | A | 0.02 | 0.017 | 0.14 | No |
| rs13090605 | 3 | 46524020 | G | 0.135 | 0.022 | 1.74E-09 | A | 0.058 | 0.036 | 0.1 | No |
| rs193106754 | 3 | 46524025 | G | -0.282 | 0.023 | 5.85E-36 | A | -0.073 | 0.036 | 0.04 | No |
| rs6773263 | 3 | 46524050 | G | -0.1 | 0.017 | 2.09E-09 | A | -0.03 | 0.016 | 0.1 | No |
| rs114127275 | 3 | 46566731 | T | 0.44 | 0.021 | 2.33E-93 | A | 0.077 | 0.026 | 0 | No |
| rs144524249 | 3 | 46575240 | C | -0.129 | 0.016 | 1.38E-15 | T | -0.03 | 0.018 | 0.13 | No |
| rs202182633 | 3 | 46608460 | ATTTTC | -0.118 | 0.016 | 2.83E-13 | A | -0.073 | 0.044 | 0.07 | No |
| rs73073714 | 3 | 46608480 | C | -0.109 | 0.018 | 1.39E-09 | T | 0.02 | 0.025 | 0.36 | No |
| rs6442008 | 3 | 46645753 | C | 0.095 | 0.017 | 1.18E-08 | T | 0 | 0.018 | 0.8 | No |
| rs2891885 | 3 | 46660344 | G | -0.125 | 0.016 | 8.74E-15 | T | 0.03 | 0.02 | 0.19 | No |
| rs12637421 | 3 | 46695074 | C | 0.161 | 0.016 | 5.43E-24 | T | -0.02 | 0.021 | 0.35 | No |
| rs80078680 | 3 | 46793763 | G | -0.15 | 0.024 | 1.81E-10 | A | 0.02 | 0.04 | 0.67 | No |
| rs116166347 | 3 | 46803291 | T | -0.174 | 0.027 | 4.63E-11 | A | 0.03 | 0.04 | 0.44 | No |
| rs7646799 | 3 | 47152567 | C | 0.1 | 0.018 | 1.54E-08 | T | -0.02 | 0.026 | 0.34 | No |
| rs35540623 | 3 | 47283357 | C | 0.101 | 0.017 | 6.10E-09 | T | -0.02 | 0.023 | 0.42 | No |
| rs12374053 | 3 | 47527859 | C | -0.139 | 0.022 | 2.95E-10 | T | -0.062 | 0.07 | 0.37 | No |
| rs4858854 | 3 | 47560006 | C | 0.092 | 0.017 | 2.95E-08 | T | -0.02 | 0.021 | 0.41 | No |
| rs2049300 | 3 | 47642726 | G | -0.125 | 0.018 | 3.11E-12 | T | -0.03 | 0.021 | 0.22 | No |
| rs12629983 | 3 | 47782356 | C | -0.119 | 0.02 | 5.25E-09 | A | -0.02 | 0.024 | 0.3 | No |
| rs75485436 | 3 | 47935009 | G | -0.343 | 0.028 | 1.07E-33 | A | -0.03 | 0.153 | 0.84 | No |
| rs12497061 | 3 | 48116168 | G | -0.1 | 0.018 | 1.80E-08 | T | 0 | 0.023 | 0.98 | No |
| rs6773254 | 3 | 48160366 | G | 0.105 | 0.018 | 3.14E-09 | A | 0.01 | 0.023 | 0.73 | No |
| rs62261476 | 3 | 48768586 | C | -0.194 | 0.023 | 1.02E-17 | T | -0.051 | 0.03 | 0.07 | No |
| rs75394422 | 3 | 49333079 | C | 0.358 | 0.041 | 1.83E-18 | T | 0.166 | 0.078 | 0.04 | No |
| rs11538155 | 3 | 49572403 | G | -0.244 | 0.032 | 4.97E-14 | T | -0.163 | 0.066 | 0.02 | No |
| rs148865391 | 3 | 49960920 | G | -0.214 | 0.033 | 6.53E-11 | A | -0.163 | 0.066 | 0.01 | No |
| rs183139656 | 3 | 50399645 | C | -0.31 | 0.041 | 3.41E-14 | T | -0.223 | 0.139 | 0.1 | No |
| rs139853420 | 3 | 50616538 | C | -0.208 | 0.034 | 1.16E-09 | T | -0.117 | 0.068 | 0.1 | No |
| rs75256143 | 3 | 52039160 | G | 0.141 | 0.024 | 3.29E-09 | G | -0.073 | 0.058 | 0.18 | No |
| rs77459111 | 3 | 52041215 | C | 0.139 | 0.024 | 4.42E-09 | T | 0.077 | 0.059 | 0.18 | No |
| rs12601721 | 17 | 32997313 | G | 0.121 | 0.018 | 9.54E-12 | G | 0.02 | 0.023 | 0.51 | No |
| rs1994089 | 17 | 33516594 | C | 0.112 | 0.016 | 5.50E-12 | T | -0.01 | 0.018 | 0.78 | No |
| rs117084209 | 17 | 33541233 | G | -0.25 | 0.043 | 6.41E-09 | G | 0 | 0.064 | 0.96 | No |
| rs117620244 | 17 | 33648381 | C | 0.353 | 0.05 | 1.87E-12 | T | -0.051 | 0.167 | 0.74 | No |
| rs4796072 | 17 | 33652270 | G | 0.115 | 0.018 | 8.12E-11 | T | 0.058 | 0.027 | 0.04 | No |
| rs62079535 | 17 | 33665329 | G | -0.238 | 0.039 | 1.05E-09 | A | -0.105 | 0.177 | 0.57 | No |
| rs113699401 | 17 | 33668796 | G | -0.147 | 0.021 | 3.35E-12 | A | 0.02 | 0.04 | 0.66 | No |
| rs8078470 | 17 | 33774159 | G | 0.097 | 0.016 | 1.05E-09 | A | -0.03 | 0.021 | 0.12 | No |
| rs72825991 | 17 | 33790987 | G | 0.189 | 0.031 | 1.07E-09 | A | -0.02 | 0.073 | 0.74 | No |
| rs148561432 | 17 | 33831939 | G | 0.27 | 0.041 | 6.78E-11 | A | 0.02 | 0.115 | 0.89 | No |
| rs72828042 | 17 | 33904832 | G | 0.352 | 0.061 | 3.22E-09 | A | -0.236 | 0.161 | 0.15 | No |
| rs225285 | 17 | 33931084 | G | -0.13 | 0.017 | 7.36E-14 | G | -0.01 | 0.023 | 0.73 | Yes |
| rs11651172 | 17 | 34270288 | G | 0.137 | 0.025 | 2.11E-08 | A | 0.03 | 0.025 | 0.27 | No |
| rs62078065 | 17 | 34287635 | C | -0.196 | 0.024 | 1.19E-16 | T | -0.02 | 0.021 | 0.31 | No |
| rs8065543 | 17 | 34341999 | G | 0.177 | 0.024 | 2.11E-13 | A | -0.02 | 0.023 | 0.4 | No |
| rs6505501 | 17 | 34347238 | C | 0.156 | 0.019 | 3.71E-16 | T | -0.02 | 0.021 | 0.36 | No |
| rs11080371 | 17 | 34375136 | C | -0.104 | 0.016 | 3.20E-10 | T | -0.01 | 0.021 | 0.61 | No |
| rs72831789 | 17 | 34396802 | T | 0.179 | 0.031 | 6.69E-09 | A | -0.041 | 0.071 | 0.62 | No |
| rs60516659 | 17 | 34403297 | G | -0.269 | 0.025 | 3.64E-27 | A | 0.02 | 0.03 | 0.6 | No |
| rs112119017 | 17 | 34443446 | C | -0.126 | 0.022 | 1.07E-08 | T | 0.077 | 0.047 | 0.12 | No |
| rs146326944 | 17 | 34818712 | G | 0.589 | 0.023 | 1.05E-147 | A | -0.041 | 0.048 | 0.34 | No |
| rs1564708 | 17 | 34825482 | C | 0.174 | 0.019 | 2.87E-20 | T | -0.03 | 0.045 | 0.54 | No |
| rs34713024 | 17 | 34840664 | G | -0.162 | 0.016 | 2.86E-23 | A | 0.02 | 0.015 | 0.18 | No |
| rs2411194 | 17 | 34877859 | C | -0.089 | 0.016 | 2.02E-08 | T | -0.01 | 0.018 | 0.74 | No |
| rs76842834 | 17 | 34883848 | C | 0.421 | 0.047 | 7.33E-19 | T | 0.095 | 0.126 | 0.45 | No |
| rs873944 | 17 | 34942072 | C | -0.242 | 0.032 | 6.59E-14 | T | 0.086 | 0.074 | 0.22 | No |
| rs17693183 | 17 | 34964290 | G | 0.58 | 0.08 | 8.93E-13 | A | -0.094 | 0.117 | 0.41 | No |
| rs9330240 | 17 | 34974689 | C | 0.475 | 0.046 | 5.84E-25 | T | 0.01 | 0.053 | 0.87 | No |
| rs975577 | 17 | 34986640 | G | 0.106 | 0.018 | 5.41E-09 | A | -0.01 | 0.021 | 0.54 | No |
| rs17138264 | 17 | 34991638 | G | -0.242 | 0.033 | 1.29E-13 | A | -0.03 | 0.058 | 0.55 | No |
| rs148883658 | 17 | 34992469 | C | 0.529 | 0.046 | 1.62E-30 | A | 0.01 | 0.1 | 0.93 | No |
| rs1867288 | 17 | 35021348 | G | -0.208 | 0.022 | 5.94E-22 | G | -0.041 | 0.032 | 0.2 | No |
| rs117990934 | 17 | 35059123 | G | -0.261 | 0.043 | 8.74E-10 | G | 0.068 | 0.081 | 0.42 | No |
| rs6607344 | 17 | 35075073 | G | -0.106 | 0.016 | 9.72E-11 | A | -0.02 | 0.023 | 0.42 | No |
| rs1252860 | 17 | 35125060 | G | -0.123 | 0.018 | 2.60E-12 | A | 0.01 | 0.02 | 0.62 | No |
| rs138066517 | 17 | 35157422 | C | -0.195 | 0.033 | 4.16E-09 | T | 0.077 | 0.057 | 0.19 | No |
| rs12450862 | 17 | 35221374 | G | -0.219 | 0.035 | 7.42E-10 | A | 0.039 | 0.056 | 0.54 | No |
| rs4795162 | 17 | 35236530 | G | -0.126 | 0.016 | 1.14E-15 | A | 0.03 | 0.02 | 0.19 | No |
| rs7213769 | 17 | 36115166 | G | -0.101 | 0.017 | 3.14E-09 | G | 0.01 | 0.033 | 0.73 | No |
| rs7221878 | 17 | 36191133 | C | 0.305 | 0.046 | 7.37E-11 | T | 0.03 | 0.047 | 0.49 | No |
| Abbreviations: CCL-4, C-C chemokine ligand 4; MIP-1b, macrophage inflammatory protein-1β; RA, rheumatoid arthritis; SNPs, single nucleotide polymorphisms. | | | | | | | | | | | |

| **Supplementary Table 4** Primary MR analyses for the association between circulating MIP-1b level and RA risk | | | | |
| --- | --- | --- | --- | --- |
| Method | Number of SNPs | OR | 95% CI | *P-*value |
| Inverse-variance weighted | 94 | 0.95 | 0.92-0.99 | 0.016 |
| Weighted median | 94 | 0.94 | 0.88-0.99 | 0.034 |
| MR-PRESSO test | 94 | 0.95 | 0.92-0.99 | 0.018 |
| MR-Egger | 94 | \ | \ | 0.859^*^ |
| ^*^*P*-value of the intercept from MR-Egger regression analysis.  Abbreviations: CI, confidence interval; MIP-1b, macrophage inflammatory protein-1β; MR, Mendelian randomization; MR-PRESSO test, MR Pleiotropy RESidual Sum and Outlier test; OR, odds ratio; RA, rheumatoid arthritis; SNP, single nucleotide polymorphism. | | | | |

| **Supplementary Table 5** The detailed information of SNPs with colocalization evidence among IVs used for MIP-1b | | | |
| --- | --- | --- | --- |
| SNP | Cytokine | *P*-value for cytokine | *P*-value for rheumatoid arthritis |
| rs11538155 | MIP-1b | 5.01E-10 | 0.016 |
| rs11574435 | MIP-1b | 1.82E-128 | 6.62E-06 |
| rs1500004 | MIP-1b | 5.30E-102 | 6.91E-06 |
| rs17765088 | MIP-1b | 4.08E-79 | 0.001 |
| rs3176953 | MIP-1b | 1.37E-106 | 4.69E-07 |
| rs41291718 | MIP-1b | 2.81E-07 | 0.015 |
| rs57093591 | MIP-1b | 6.92E-110 | 2.40E-06 |
| rs73062378 | MIP-1b | 1.31E-23 | 0.044 |
| rs73075560 | MIP-1b | 1.11E-48 | 0.003 |
| rs9817966 | MIP-1b | 1.95E-40 | 0.008 |

Abbreviations: IV, instrumental variable; MIP-1b, macrophage inflammatory protein-1β; SNPs, single nucleotide polymorphisms.
